# An open-source, externally validated neural network algorithm to recognize daily-life gait of older adults based on a lower-back sensor

**DOI:** 10.1007/s11517-025-03466-z

**Published:** 2025-10-22

**Authors:** Yuge Zhang, Sjoerd M. Bruijn, Michiel Punt, Jorunn L. Helbostad, Mirjam Pijnappels, Sina David

**Affiliations:** 1 Department of Human Movement Sciences, Faculty of Behavioural and Movement Sciences, Amsterdam Movement Sciences, Vrije Universiteit Amsterdam, Van Der Boechorststraat 7, 1081 BT Amsterdam, The Netherlands; 2 Research Group Lifestyle and Health, Utrecht University of Applied Sciences, Utrecht, The Netherlands; 3 Department of Neuromedicine and Movement Science, Faculty of Medicine and Health Sciences, Norwegian University of Science and Technology, Trondheim, Norway

**Keywords:** Walking, Deep learning, Classification, Aged, Digital health

## Abstract

Gait recognition is critical for daily-life fall risk assessment and rehabilitation monitoring, but existing models face many challenges that limit their use in daily life for older adults. We aimed to develop an open-source gait recognition for older adults using sensor data and explore the effect of data augmentation on model training. A convolutional neural network was trained using lower-back inertial sensor data from 20 participants (mean age 76.4) and externally validated on 47 participants (mean age 72.3). The model was trained using 6-channel data (accelerations and angular velocities) and 3-channel data (accelerations only), with and without data augmentation. On the testing dataset, the best 6-channel model achieved accuracy of 91.4%, precision of 59.7%, sensitivity of 99.5%, F1-score of 74.7%, and specificity of 90.3%, and the best 3-channel model achieved accuracy of 96.5%, precision of 78.7%, sensitivity of 98.9%, F1-score of 87.6%, and specificity of 96.1%. On the external validation dataset, the best models with both channels show near-perfect scores. This study demonstrates that the convolutional neural network algorithm based on lower-back inertial sensor data can accurately recognize daily-life gait of older adults, and data augmentation was especially beneficial for models using acceleration data only.

**Graphical abstract:**

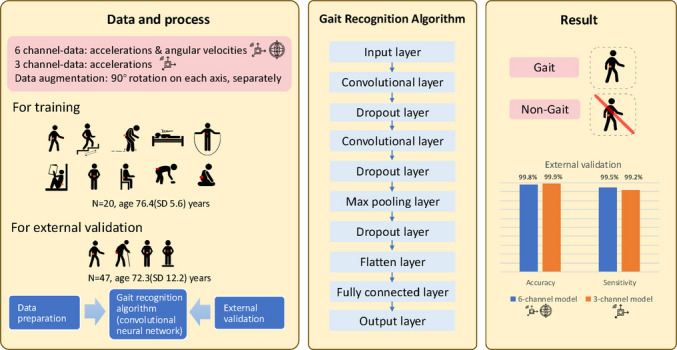

**Supplementary Information:**

The online version contains supplementary material available at 10.1007/s11517-025-03466-z.

## Introduction

Each year, approximately one-third of older adults experience falls [1, 2], most of which occur during walking [3]. Many studies have shown the potential of gait measures derived from inertial measurement units (IMUs) to predict fall risks [4, 5], and rehabilitation monitoring, such as for stroke survivors [6]. However, to extract these measures, the first crucial step is to recognize gait among various daily-life physical activities accurately is necessary and crucial.

Convolutional neural networks (CNN) can learn features via filters (or kernels) and have been increasingly used for gait recognition on IMU data [7]. Most of the developed models are based on young adults and their accuracy in recognizing gait is greater than 88% [8, 9]. However, older adults typically walk slower or suffer from gait impairments, thus models trained on young adults may not be suitable for older adults.

Several challenges must be recognized and addressed when applying such models to older adults.

First, the placement of the IMU sensor must be both practical—ensuring user adherence—and capable of providing reliable data for detecting gait episodes. While wrist-worn sensors may be preferred by most users, as they can be integrated into a watch, their reliability in detecting gait episodes is mediocre [10]. Consequently, sensors worn on the lower back or waist are generally considered more effective for gait recognition tasks.

Second, the data collection conditions affect the model’s robustness. Most studies used data that were collected in a structured protocol in the lab [11, 12] or in semi-structured conditions. In the semi-structured conditions, activities can be performed out of sequence, labeled by the experimenter in a simulated environment, or by participants themselves using an electronic device in a daily life condition [13–15]. However, such activities are different from daily life in context, behavior, and rhythm [16], and especially when there is an observer on site, activities might be influenced by the Hawthorne effect [17]. Because of these challenges, unsupervised data with valid activity labels collected in daily life is lacking. One of the few exceptions is the data set of Bourke and colleagues who collected an IMU dataset (the ADAPT dataset) of older adults, including standardized data in semi-structured activities, and data in daily-life conditions [18], labeled by synchronized video footage from a body-worn camera.

Third, daily-life data is often imbalanced in activities with a smaller amount of gait. Therefore, sensitivity and F1-score are important to evaluate the gait recognition model. Moreover, to evaluate the robustness of a trained model, external validation of the model is necessary. As an example, in the study of Zou [9], the CNN model achieved an accuracy of 92.9% for gait on their training dataset but only 40.6% on the external dataset, showcasing the importance of testing and reporting results for external validation data. Third, to increase the robustness of models, the variability of the data should be considered. Data augmentation (DA) can expand a dataset synthetically by transforming existing samples, increasing the training data's quantity and variability [19]. Um and colleagues found that adding rotated data can alleviate the effect of variability of sensor orientations, thereby improving accuracy by 11.9% on the testing dataset [20].

Finally, only a few studies have made their algorithms available in an easy-to-use software repository [9, 21], which limits their accessibility, practical application, and potential impact. Therefore, to overcome all these challenges, we aimed to develop an open-source externally validated neural network algorithm for recognizing daily-life gait based on lower back IMU data of older adults. Additionally, we will explore the effects of sensor rotation as a data augmentation technique on the model based on the 3-dimensional accelerations (3-channel IMU data) only or on a combination of the 3D accelerations and 3D angular velocities (6-channel IMU data).

## Methods

### Datasets

We used two datasets: one was the ADAPT dataset for model training [18] and the second one was a dataset of stroke survivors serving as external validation [22, 23]. The data description and the code belonging to this paper (including the trained CNNs) are available at [24]. Details of the participants’ characteristics are presented in Table 1.

**Table 1:** Demographic characteristics of participants

Characteristics	ADAPT	Balance tests in External dataset	Walking tests in External dataset
Number	20	47	18
Age (years) (Mean (SD))	76.4 (5.6)	72.3 (12.2)	68.4 (9.3)
Sex (Male/Female)	5/15	30/17	12/6
Height (m) (Mean (SD))	1.67 (0.07)	1.75 (0.11)	1.74 (0.12)
Weight (kg) (Mean (SD))	73.7 (11.4)	78.2 (14.5)	79.7 (15.4)
Participants with disabilities/diseases that affect activity (%)	60	100	100
Hemiparetic side (left/right/both/unknown)	N.A	26/10/8/3	8/4/5/1
Stroke types (ischemic/hemorrhagic/subarachnoid)	N.A	38/6/3	14/3/1/0
Gait speed (m/s) (Mean (SD))	N.A	0	1.3 (0.6)
Walking bout (s) (Median (IQR)	4.1 (4.5)	0	120 (0)
Participants with falls in the past year (%)	55	N.A	N.A

*SD* Standard deviation; *IQR* Interquartile Range, the difference between the 75th and 25th percentiles of the data

#### Training dataset

The ADAPT dataset is a comprehensive physical activity reference dataset, for which 20 older adults performed various activities in semi-structured and daily-life environments, wearing 12 body-worn IMUs and a synchronized chest-mounted GoPro camera [18]. We were primarily interested in the lower-back sensor data as the clinical value of the lower back for trunk stability, balance control, and fall risk assessment [25]. The data was collected by the Usense (University of Bologna, Italy) sensor at a sampling frequency of 100 Hz. All participants were able to take verbal instructions and walk independently for 100 m without a walking aid. Twelve participants had self-reported disabilities or diseases affecting activity (unspecified). Eight participants have experienced one or more falls in the past year.

This dataset contained 43.9 h of activities in total, including 9.5 h in semi-structured conditions and 34.4 h in daily-life conditions. All activities were annotated according to strict activity definitions based on the video footage [26]. There were 16 labeled activities: walking, walking with transitions, shuffling, standing, sitting, transition, leaning, jumping, dynamic, static, lying, shaking, picking, kneeling, stair ascending, and descending. Walking was defined as locomotion towards a destination with one stride or more, finishing with one foot placed beside the other foot, also including certain steps during the transition from walking to another posture [26]. There were 6.13 h of walking in total, accounting for 14% of all activities, and the agreement of video labels by raters was > 90% [26]. For model training, all activities not labelled as walking were categorized as non-walking. For model training, all activities not labeled as walking were categorized as non-walking. This was done because the primary task of the developed algorithm is to identify gait episodes, which are of particular interest for monitoring. Furthermore, reducing the number of labels is expected to improve the algorithm’s performance [27, 28]. Each participant’s distribution of walking bouts is shown in Supplementary Fig. 1.

#### External validation dataset

The external validation data was collected by Felius and colleagues [22, 23] from 47 stroke survivors wearing an inertial sensor (Aemics b.v. Oldenzaal, The Netherlands) sampling at 104 Hz on the lower back (L5). This dataset provided the opportunity to externally validate the algorithm on a different population, using a different sensor and collected in a different environment. Data from balance tests and walking tests were categorized as non-walking data and walking data, respectively. All participants could independently complete balance tests, and 18 of them could independently walk. To make it consistent with the ADAPT data, in this study, we used the data where participants walked without using aids. For completeness, we also provided the information of participants who walked with aids, shown in Supplementary Table 2.

The balance tests included one condition of sitting and four conditions of unsupported standing (eyes open, closed, feet together, and foam). The walking test was conducted on a 14-m-long surface walkway for two minutes, including turns at both ends [23]. Participants walked at a self-selected pace without stopping, being distracted, or developing observable clonus. As a calibration, participants remained stationary before and after the tests for a period.

### Data processing

**Fig. 1:**
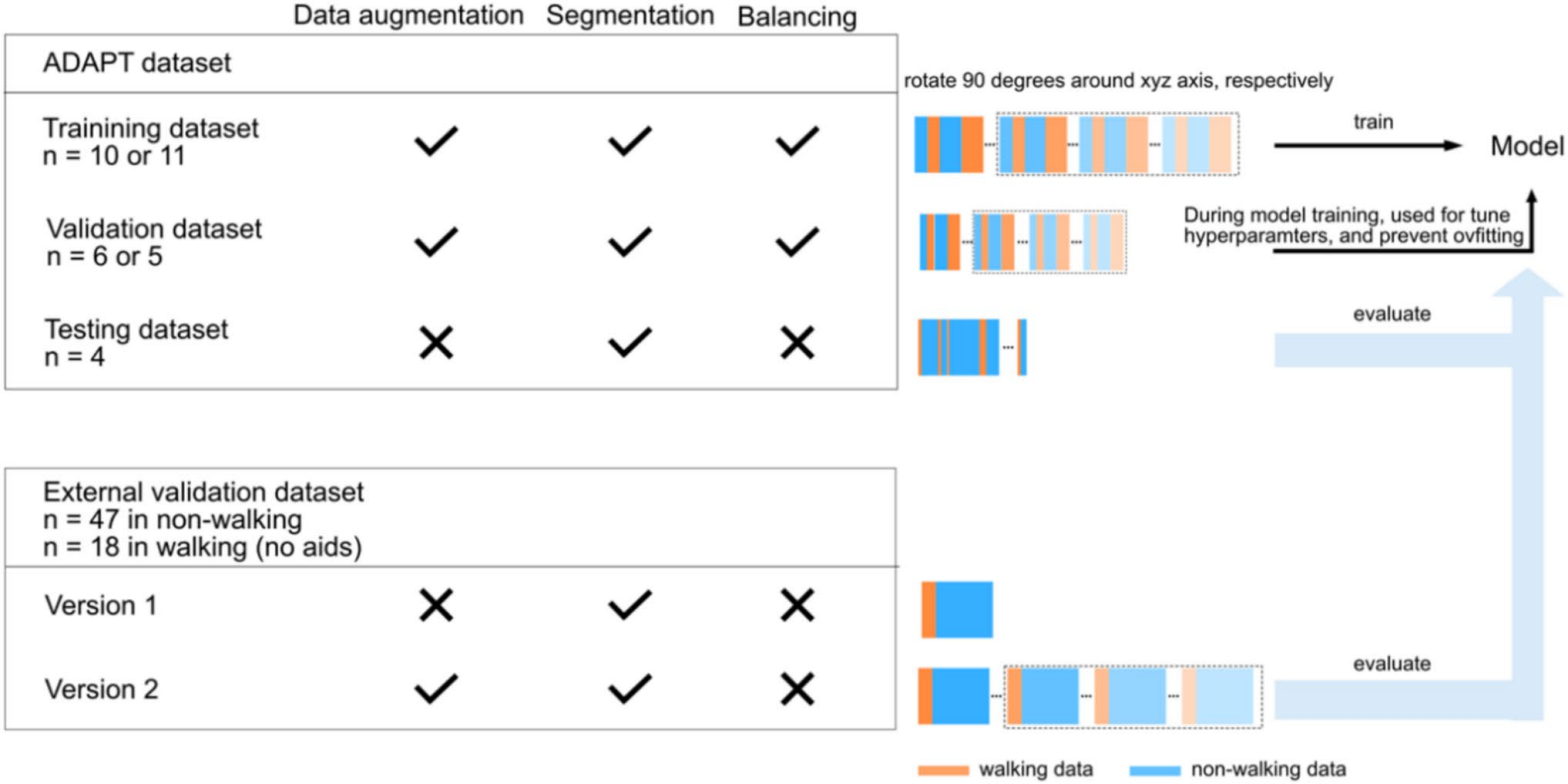
The data process for model training and evaluation

Figure 1 gives an overview of the data processing for model training and evaluation. For model training, the steps included data preparation, splitting, augmentation, segmentation, and balancing. For the external validation, steps included data preparation, augmentation, and segmentation. We used the input of six channels (acceleration and angular velocity) and three channels (acceleration only) separately. Matlab 2021 and Python (version 3.8) were used.

#### Data preparation

The ADAPT dataset was organized as six-channel IMU data, activities labels, and the participant’s ID, resulting in a 16007994 × 6 input matrix, a 16007994 × 1 target matrix, and a 16007994 × 1 group matrix, respectively. For the external dataset, after deleting the calibration data, the dataset was organized as IMU data, labels, and participants’ ID, resulting in a 2523115 × 6 input matrix, 2523115 × 1 target matrix, and a 2523115 × 1 group matrix, respectively.

#### Data splitting

We used a repeated hold-out validation method to split the ADAPT dataset for model training. Each time, the dataset was randomly divided by participants, resulting in the percentage of training, validation, and testing datasets as 55%, 25 and 20%.

#### Data augmentation (DA)

The training and validation datasets from ADAPT were augmented by applying 90° rotation to each signal channel to simulate major sensor placement shifts. A 90° rotation changes the main movement axis, helping to ensure that the algorithm does not rely on one specific channel for learning. For comparison, we also trained the model without data augmentation. To increase the sample size with different sensor orientations, the external dataset was augmented as well.

#### Data segmentation

Studies indicate that smaller windows were preferable for low-intensity activities and older adults [29]. An interval of 1–2 s seems the best trade-off between accuracy and recognition time [30, 31]. Therefore, before being fed into the model, all datasets including the external validation data were segmented into 2-s windows with 50% overlap, with each window assigned a single activity label. Additionally, in each window, data was normalized by subtracting the corresponding mean value on each axis.

#### Data balancing

The dataset for model training was imbalanced, containing 15235 walking windows and 131208 non-walking windows (a rate of 1:8.61). Such imbalances can bias model performance [32, 33]. To address this, for both the training and validating datasets, we down-sampled the number of non-walking windows to the same as the number of walking windows. The testing and external validation datasets were not down-sampled to maintain the uncertainty and diversity of data. The number of walking windows in the external validation dataset was 11,271 and the number of non-walking windows was 89139, indicating a rate of 1:7.91.

### CNN model structure

The structure of the CNN model developed in this study contains the following layers.

1. An input layer. The partitioned signal data fed into the first layer was a 3D matrix form of (batch size × windows × number of channels), where batch size B = 32, sliding window size W = 200 (which resembles 2 s), and the number of channels *N* = 6 (i.e. acceleration and angular velocity data), or *N* = 3 (i.e. acceleration only).
2. Hidden layers, including two convolutional layers, one max pooling layer, and one fully connected layer. A convolution layer with multiple filters creates multiple feature maps, enabling the extraction of a diverse set of features from the input [34]. Here, we used 64 filters, so that each convolutional layer has 64 feature maps from the previous layer. The weights of the filters are automatically updated in subsequent training iterations (epochs). The max pooling layer reduces model parameters by maintaining the maximum values of each feature map. The fully connected layer converts the multidimensional array from the previous layer to a single-dimensional vector.
3. Dropout layers. Although the representational ability of deep neural networks is compelling, overfitting remains a significant drawback due to the non-linear hidden layers [35, 36]. By randomly setting the output of a portion of the neurons to zero, using dropout in the training process reduces the neuron dependencies and significantly improves the generalization ability. In our study, a dropout layer with a rate of 50% [37] was added after each convolutional and pooling layer.
4. The output layer. This layer leverages features from the fully connected layer to generate the probability distribution of the categories, generally adopting the ‘Softmax’ function.

To prevent the network from overfitting, we also used ‘Early Stopping’ to stop training when the loss value did not improve for 3 consecutive epochs. To evaluate model performance, we calculated the accuracy, precision, sensitivity, F1-score, and specificity between predicted labels and true labels for the validation, testing, and external validation datasets.

During the repeated hold-out validation process, the best-trained model was selected based on the highest sensitivity performance, reflecting the model’s ability to correctly identify all relevant classes.

## Results

**Fig. 2:**
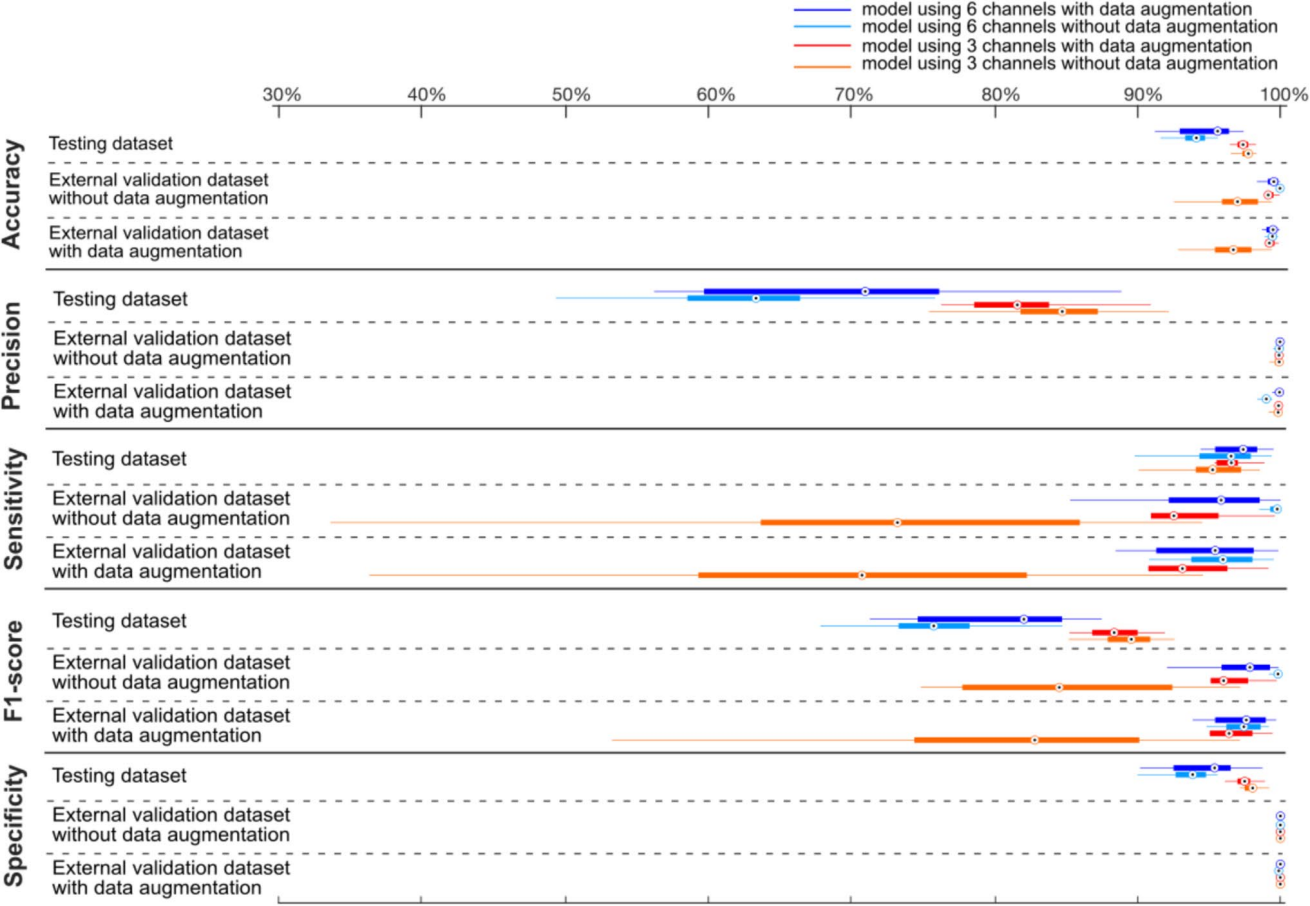
The median (IQR) of model performance over multiple training times (DA: data augmentation; IQR: the Interquartile Range, the difference between the 75th and 25th percentiles of the data)

Figure 2 presents the median results (with interquartile range, IQR) of model performance after training our models on different dataset splits. Detailed values are shown in Supplementary Table 1, which also includes results of the external datasets using walking aids.

For the testing dataset, the range of median values of performance across all types of models were [94.1%, 97.8%] for accuracy, [63.4%, 84.8%] for precision, [95.3%, 97.4%] for sensitivity, [75.8%, 89.6%] for F1-scores and [93.9%, 98.1%] for specificity. For the testing dataset, the 3-channel model outperformed the 6-channel model in terms of accuracy, precision, F1-score, and specificity, while showing comparable sensitivity (Fig. 2), no matter whether using DA or not. In addition, using DA in the 6-channel model increased the precision and F1-score by 7.6% and 6.3%, while having little influence on its accuracy, sensitivity, and specificity.

For the external validation dataset, the range of median values of performance across all types of models was [97%, 100%] for accuracy, [99.9—100%] for precision, [73%, 100%] for sensitivity, [85%, 100%] for f1-scores, and 100% for specificity. In contrast to the testing dataset, in the external validation dataset, the 6-channel model performed generally better than the 3-channel model. In addition, using DA had little influence on the 6-channel model but greatly increased the median sensitivity on the 3-channel model i.e., from 70. 8% to 93.2% in external validation data with augmentation, from 73.2% to 92.5% in the raw external validation data. Also, it increased the median F1-score on the 3-channel model, by 13.6% and 11.5% on external validation data with and without DA, respectively.

Furthermore, in Supplementary Table 1, we showed the performance on the external validation for the participants who used walking aids. We found the 6-channel model performed still well when walking with aids, as the median sensitivity ranged from 73 to 89%. However, the 3-channel model without data augmentation performed worst with a median range of sensitivity of 48%, after using data augmentation, the value greatly increased to 66%.

Table 2 shows the performance of the best-trained models using different channels as input. The best 6-channel model was trained without DA, as it achieved perfect results during external validation. This model performs with the accuracy, precision, sensitivity, F1-score, and specificity of 93.3%, 60.6%, 99.2%,75.3%, and 92.7% on the testing dataset. The best-trained 3-channel model was trained using DA, with the accuracy, precision, sensitivity, F1-score, and specificity of 96.5%, 78.7%, 98.9%,87.6%, and 96.1% on the testing dataset. For both channels, the external validation results (with and without DA) show near-perfect scores, particularly without DA. These two models can be found in easy-to-use functions [24].

**Table 2:** Summary of gait recognition performance of the best models

Training model on 6 channels without DA					
Dataset	**Accuracy**	**Precision**	**Sensitivity**	**F1-score**	**Specificity**
Testing	91.4%	59.7%	99.5%	74.7%	90.3%
External validation with DA	99.9%	99.5%	99.8%	99.7%	99.9%
External validation without DA	100.0%	99.8%	99.9%	99.8%	100.0%
Training model on 3 channels with DA					
Dataset	**Accuracy**	**Precision**	**Sensitivity**	**F1-score**	**Specificity**
Testing	96.5%	78.7%	98.9%	87.6%	96.1%
External validation with DA	99.9%	99.7%	99.2%	99.4%	100.0%
External validation without DA	99.9%	99.9%	99.6%	99.7%	100.0%

## Discussion

We developed an open-source, externally validated CNN model to recognize daily-life gait in older adults and explored the effect of the use of data augmentation in model training. For the model with 6-channel input data (both acceleration and angular velocity), the overall accuracy exceeded 93.3%, with precision over 60.6%, sensitivity over 98.7%, F1-score over 75.3%, and specificity over 90% across testing and external validation datasets. For the model with 3-channel input data (acceleration only), the overall accuracy was over 96.4%, with precision over 78.7%, sensitivity over 97.2%, F1 score over 87.6%, and specificity over 95.5%.

In the testing dataset, the 3-channel model performed better than the 6-channel model in accuracy, precision, F1-score, and specificity, except for the sensitivity, which was similar. This was not consistent with other studies that showed that adding angular velocities can be complementary to increasing the model’s sensitivity [9, 32, 33, 38]. The slight drop in precision and recall for both the 3- and 6-channel models on the test dataset (Fig. 2, Table 2) may be indicative of overfitting. To mitigate this during model training, we implemented early stopping to prevent the model from over-specializing on the training data. However, without a more comprehensive external validation dataset, definitive conclusions cannot be drawn. Increasing the training dataset size may further reduce the risk of overfitting.

In addition, data augmentation had little influence on training the 3-channel model (median accuracy 97.4% vs 97.8%, precision 81.6% vs 84.8%, sensitivity 96.6% vs 95.3%, F1-score 88.4% vs 89.6%, specificity 97.5% vs 98.1%, for with and without DA), but increased the precision and F1-score in the 6-channel model (71.0% vs 63.4%, 82.1% vs 75.8% for with and without DA). This indicates that such data augmentation may prove to be especially important when only accelerometer data (as opposed to accelerometer and gyroscope data) is used, as accelerometer data alone may not capture gait fully.

In the external validation dataset, the accuracy of models was greater than 94%, which is a huge improvement when compared to the 40.6% reported by Zou et al. [9] on the external validation data collected in structural conditions. Additionally, the 6-channel model performed better than the 3-channel model, which is in line with other studies [9, 32, 33, 38]. Using data augmentation had little influence on the 6-channel model but greatly increased the median sensitivity and F1-score of the 3-channel model (in the 6-channel model, 95.8% vs 99.8%, 97.9% vs 99.8%, for with and without DA; in the 3-channel model, 92.5% vs 73.2%, 96.0% vs 84.5%, for with and without DA). This suggests that data augmentation indeed had added value for 3-channel data.

Although both testing and external validation datasets were imbalanced, there were three main differences between them, which may cause the different performances. Firstly, the testing dataset was collected in daily-life conditions, and the external data was collected in a structured condition. Due to this, in the testing dataset, for each participant’s data, gait was alternated with other activities, such as stand-walk-sit and sit-walk-stairs. However, in the external validation dataset, because gait and non-gait were dispersed in data collection, the same activity was pooled across all subjects. Meanwhile, the daily-life environment may also alter gait patterns, e.g., slopes and obstacles may affect how people walk, and people may walk differently indoors and outdoors [25]. Therefore, activity context may significantly influence the model performance [16], as evidenced by the overall better performance on the structured external dataset than on the daily-life testing dataset. On the other hand, this also indicates that a model trained on daily-life data can also perform well on structured data.

Secondly, the testing dataset had various non-gait activities, such as shuffling, walking with transitions, and going up and down the stairs. The external dataset included only two static activities, which were sitting and standing. Studies showed that the sensor data of dynamic activities, such as going up and down stairs, may be more easily misclassified as gait [39, 40]. Therefore, the non-gait activities in the testing dataset were easier to distinguish so the model performance was better.

Lastly, studies showed that a short walking duration and slow walking speed negatively affect the model performances [25, 41]. In the testing dataset, as shown in the Supplementary Fig. 1, most walking bouts were between 2 s and 6.45 s (median + IQR/2), and the longest walking bouts were shorter than 170 s. Because parts of these bouts are needed to accelerate and decelerate [42], the walking speed of the testing dataset can be expected to be relatively slower than observed in the external dataset. On the contrary, in the external data, even though participants were stroke survivors, they walked independently with an average speed of 1.3 m/s in a 2-min walkway (calculated from the IMU data) [23]. Such a situation may also explain why models performed better on the external validation dataset than the testing dataset. Van Ancum demonstrated that the 4-m gait speed is only related to the high end of daily-life speed and cannot reflect the walking ability of daily life [43]. Therefore, further studies should collect more daily-life data indoors and outdoors for training and external validation, to cover various walking speeds, walking distances, and contexts [38].

The results on walking using aids showed similar results as for walking without aids, even though our models were trained on only walking without aids, suggesting that our models are applicable to a wide range of gaits.

### Strengths and limitations

The model we developed can correctly recognize the daily-life gait of older adults and was externally validated on stroke survivors. In addition, we provided the model for different input channels and explored the effect of the use of data augmentation in the model training. Last but not least, we offer the best models and code for step-by-step data processing to obtain gait episodes from either 3 or 6-channel data. Users can directly use and refine our models from the GitHub repository [24].

There are also some limitations of our study. Firstly, the external dataset was structured, which may have inflated model performance. Thus, model performance on an external daily-life dataset is unclear yet. Future work requires an unstructured external validation dataset comprising multiple activities of daily living to better assess model performance. Secondly, we used down-sampling methods to balance our training data, thereby reducing the number of available training windows. More importantly, the training data in our study consisted of only 10 or 11 participants, insufficient to cover the varied gait patterns of a wide population of older adults, thus, increasing the number of participants for training is recommended to improve the robustness. Lastly, in this study, we used 50% overlapping windows. Further studies can try to use an overlap of 95%−99% and then use approaches like majority voting machine learning for a more accurate model prediction.

## Conclusions

In conclusion, this study provided a good convolutional neural network for daily-life gait recognition based on lower-back IMU data for healthy and gait-impaired older adults, available on (https://github.com/ZYuge/Recognize-real-world-walking-based-on-deep-learning-methods). Besides, we found when training the model, the use of data augmentation is especially helpful on the model based on acceleration data only. The open-source code promises to promote community-based remote gait monitoring.

## Supplementary Information

Below is the link to the electronic supplementary material.

Supplementary File 1 (DOCX 95.5 KB)

## Data Availability

The open-source software tool can be accessed online at https://github.com/ZYuge/Recognize-real-world-walking-based-on-deep-learning-methods.
